# Asymmetric effects of spectator presence on home and away team performance: a natural experiment from the 2021 K League 1 season

**DOI:** 10.3389/fpsyg.2025.1646264

**Published:** 2025-09-01

**Authors:** Joonha Lee, Seoung-Jin Park, Dong Hun Suh, Hyung Jun Park

**Affiliations:** ^1^Department of Orthopedic Surgery, Yeson Hospital, Bucheon, Republic of Korea; ^2^Department of Orthopedic Surgery, Gachon University College of Medicine, Incheon, Republic of Korea; ^3^Department of Orthopedic Surgery, Soonjung Hospital, Suncheon, Republic of Korea; ^4^Department of Orthopedic Surgery, Korea University Ansan Hospital, Ansan, Republic of Korea

**Keywords:** spectator effect, home advantage, natural experiment, match performance, professional football

## Abstract

**Introduction:**

The presence of spectators is a well-known factor contributing to home advantage in competitive sports. However, isolating its direct effect on team performance has proven difficult. This study aims to assess the influence of spectator attendance on the performance of home and away teams using a natural experimental setting during the 2021 K League 1 season, which experienced regional differences in spectator policies due to the COVID-19 pandemic.

**Methods:**

This retrospective observational study analyzed 162 matches in the 2021 K League 1 season. Match variables were categorized into three domains: outcome factors (e.g., points, win percentage), tactical factors (e.g., shots on target, total shots), and violation factors (e.g., fouls, cards) to evaluate team performance. Matches were classified according to the presence or absence of spectators and further stratified by whether they were home or away games. Group comparisons were performed using t-tests, one-way analysis of variance, and effect-size analysis (Cohen’s *d*).

**Results:**

The presence of spectators was associated with improved tactical performance in home matches, as evidenced by a greater number of shots on target (6.02 ± 3.08 vs. 4.40 ± 2.34, *p* = 0.041, *d* = 0.58) and total shots (11.46 ± 4.13 vs. 9.46 ± 3.48, *d* = 0.52). In contrast, spectator-related effects were more pronounced in away matches, particularly in outcome performance factors. Away teams with spectators had lower win percentage (0.22 ± 0.42 vs. 0.58 ± 0.52, *d* = 0.83), more goals conceded (1.58 ± 1.16 vs. 0.67 ± 0.89, *p* = 0.032, *d* = 0.82), and fewer points (0.96 ± 1.19 vs. 2.00 ± 1.28, *d* = 0.87) than those without spectators.

**Conclusion:**

Spectator presence was associated with enhanced tactical performance by home teams. In contrast, its impact on away matches was more pronounced in outcome-related performance, suggesting that home crowds may exert psychological pressure that disrupts visiting teams’ execution and composure. These findings underscore the asymmetrical influence of spectatorship and enhance our understanding of home advantage in professional football.

## Introduction

Spectator attendance is widely recognized as a crucial determinant of home advantage in competitive sports (Courneya and Carron, 1992; Nomura, 2022; Cross and Uhrig, 2023; Wang and Qin, 2023). Surveys involving diverse stakeholder groups, including fans, athletes, coaches, and referees, have consistently identified crowd support as the most influential contributor to home advantage (Nomura, 2022). The effects of spectatorship have been documented across numerous sports, including football, rugby, basketball, handball, and even in individual disciplines such as skeleton racing (Chun and Park, 2020; Fioravanti et al., 2021; Fischer and Haucap, 2021; Gershgoren et al., 2021; van Bommel et al., 2021; Nomura, 2022). These effects tend to be more pronounced in amateur-level competitions and events with larger audiences, suggesting a dose–response relationship between crowd size and performance outcomes (Fischer and Haucap, 2021; Nomura, 2022). Schwartz and Barsky et al. identified three primary mechanisms underlying home advantage: spatial familiarity, reduced travel-induced fatigue for the home team, and crowd support. Among these, crowd presence is uniquely susceptible to external modulation (Schwartz and Barsky, 1977). It may bolster player confidence and concentration while simultaneously exerting psychological pressure on referees, thereby influencing officiating behavior (Loughead et al., 2011; Goumas, 2014a, 2014b; Ponzo and Scoppa, 2016). Nonetheless, the isolated effect of crowd presence remains challenging to assess empirically, as it is rarely subject to controlled variation in a real-world setting (Loughead et al., 2011; Goumas, 2014a, 2014b; Ponzo and Scoppa, 2016). The COVID-19 pandemic, which emerged in late 2019, provided an unprecedented opportunity to overcome this methodological barrier. Widespread restrictions on public gatherings led many professional leagues to conduct matches without spectators (Huang et al., 2020; Yasutaka et al., 2021; Tamir, 2022). Several studies have sought to capitalize on this natural experiment by comparing matches played before and during the pandemic (Grazioli et al., 2020; Mon-Lopez et al., 2020; Hill and Van Yperen, 2021; McCarrick et al., 2021; Moreno-Perez et al., 2021; Scoppa, 2021; Sors et al., 2021; Wunderlich et al., 2021). However, most studies were constrained mainly by inter-seasonal comparisons and vulnerable to potential confounding factors, such as player turnover, tactical shifts, and psychological adaptations to pandemic conditions (Grazioli et al., 2020; Mon-Lopez et al., 2020; Hill and Van Yperen, 2021; McCarrick et al., 2021; Moreno-Perez et al., 2021; Scoppa, 2021; Sors et al., 2021; Wunderlich et al., 2021; Cross and Uhrig, 2023). Consequently, these studies struggled to isolate the pure effect of spectatorship, leaving uncertainty regarding its true contribution to match outcomes.

Within this context, the 2021 season of South Korea’s professional football league, K League 1, offers an exceptional quasi-experimental design for examining the effect of spectatorship under more controlled conditions. Although the 2020 season was delayed and conducted without spectators because of public health concerns (Yoon, 2021), the 2021 season resumed its conventional spring-to-winter schedule, facilitated by improved epidemic control and nationwide vaccination efforts (Yoo, 2021a). This continuity was made feasible through the Korean government’s region-specific strategy, which eschewed nationwide lockdowns in favor of targeted social distancing policies calibrated to local epidemiological trends (Koh, 2020; Lim et al., 2021; Steyn et al., 2021). Nevertheless, a surge in COVID-19 cases driven by the delta variant in July 2021 necessitated renewed restrictions in the Seoul Capital Area, including Seoul, Incheon, and Gyeonggi province. Consequently, the five teams in this region were required to play home matches without spectators. By contrast, the remaining seven teams outside the capital area maintained spectator attendance for most of their home matches (Jung, 2021). This intraseasonal divergence in spectator policy, driven by regional public health mandates, created a natural experiment in which the presence or absence of a crowd was systematically determined by geographic location rather than team characteristics (Yoo, 2021b; Yun, 2021; Kim et al., 2022). Moreover, public health regulations permitted only home-team supporters to attend the event. Away fans were strictly prohibited, creating a more controlled setting to isolate the effects of partisan crowd presence without interference from opposing or neutral spectators.

Given this unique context, we aim to determine whether the presence of spectators influences team performance during professional football matches. Furthermore, we examined whether the magnitude of this influence differed between the home and away teams. We hypothesized that the presence of spectators would enhance home team performance. Additionally, we hypothesized the impact of spectators would be more pronounced for away teams than for home teams, given their increased psychological vulnerability in hostile environments. This setting aligns particularly well with the framework of Schwartz and Barsky et al., wherein crowd support is posited as a central component of home advantage (Schwartz and Barsky, 1977). Given that venue familiarity and travel fatigue were relatively constant across teams, our design facilitates a focused examination of crowd support as a distinct causal mechanism.

## Materials and methods

### Inclusion and exclusion criteria

**Figure 1:**
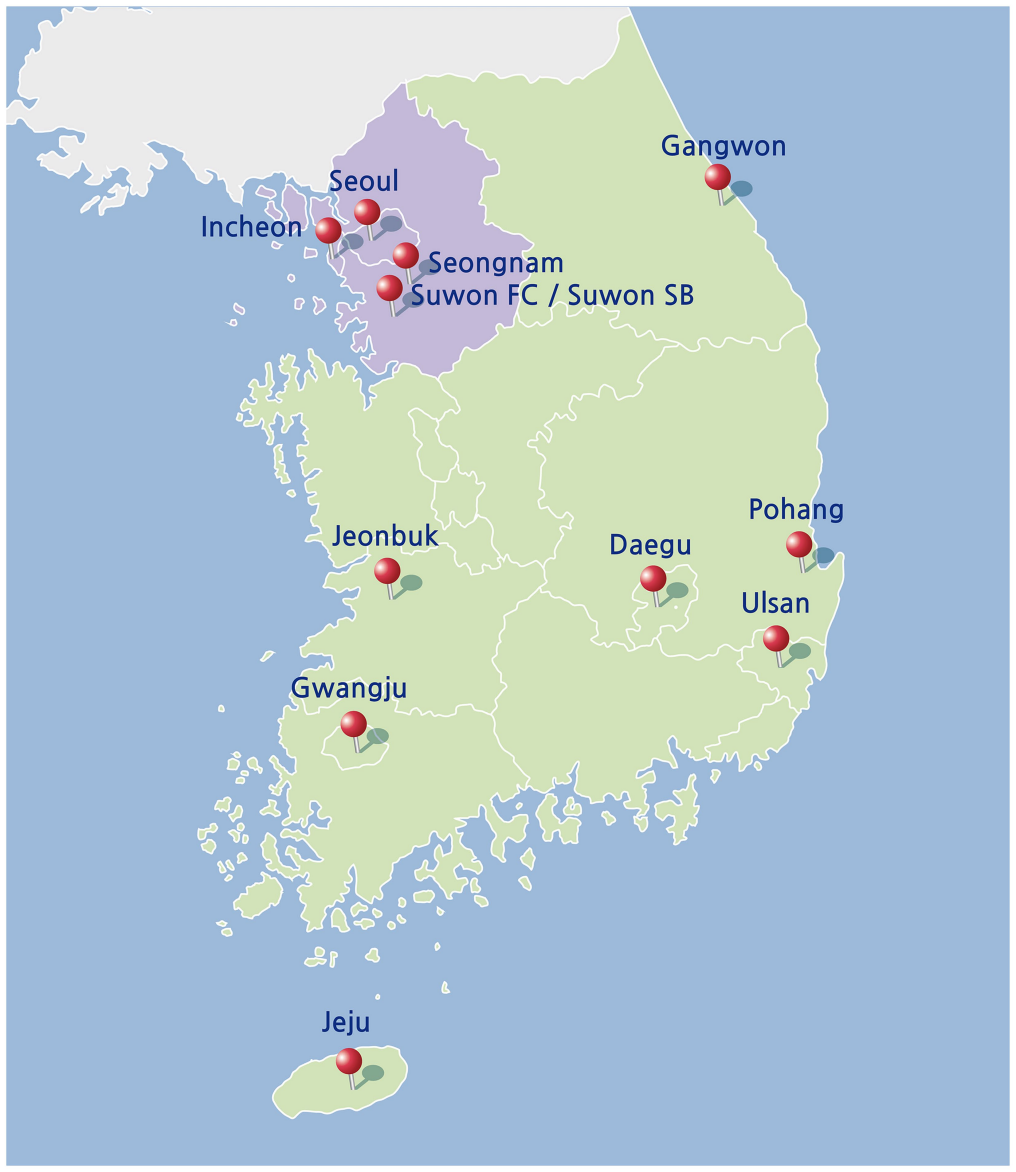
Geographical distribution of K League 1 teams in the 2021 season.

The 2021 season of K League 1 took place from February 27 to November 5, involving 12 professional football clubs. Among them, five clubs based in the Seoul Capital Area — Incheon United, Seongnam FC, FC Seoul, Suwon FC, and Suwon Samsung Bluewings — were subject to stricter and regionally variable spectator policies throughout the season. Under local public health guidelines, these teams were permitted to admit a limited number of home spectators from the beginning of the season through late June. However, following a regional surge in COVID-19, all home matches in the Seoul Capital Area were conducted without spectators from July 1 through October 22. Limited spectator access was reinstated on October 23. In contrast, the seven clubs located outside the capital region—Jeonbuk Hyundai Motors, Ulsan Hyundai, Daegu FC, Pohang Steelers, Gangwon FC, Gwangju FC, and Jeju United—were allowed to host matches with restricted home crowd attendance throughout the entire season without any whole suspension period. Under nationwide public health regulations, only local home-team supporters were permitted to attend matches. Away spectators were strictly prohibited at all venues and at all times. To ensure analytical consistency, match classification was conducted from the perspective of the five teams based in the Seoul Capital Area. Consequently, a greater number of away matches featured spectators, as non-capital region teams were permitted to host crowds throughout most of the study period. Home matches played by Seoul Capital Area teams were categorized as either “home matches without spectators” or “home matches with spectators,” based on the presence of a crowd. Likewise, their away matches were classified into two groups—“away matches with spectators” and “away matches without spectators”—according to whether spectators were present at the host venue (Figure 1).

Matches from the final round of the K League 1, held after October 30, were excluded from the analysis because of structural imbalances in team strength. The final round adopted a split-format system, comparable to that of the Scottish Premiership (Reilly and Witt, 2021), in which the league was divided into two groups (top six and bottom six) after 33 rounds, followed by five additional intra-group matches (K League, 2018). Given the inherent imbalance in team rankings at this stage, these matches were deemed unsuitable for performance comparisons.

The final analysis included 162 games: 35 home matches without spectators, 48 home matches with spectators, 12 away matches without spectators, and 67 away matches with spectators. There was no statistically significant difference in the mean pre-match ranking of opponents across the four groups, indicating that the team matchups had comparable competitive strength (Table 1).

**Table 1:** Average standings of the opponents of games in each group.

Variables	Home matches		Away matches		*p* value	*Post-hoc* significance
	Without spectators (*n* = 35)	With spectators (*n* = 48)	Without spectators (*n* = 12)	With spectators (*n* = 67)		
Rank of the opposite teams	6.97 ± 3.47	6.54 ± 3.22	6.75 ± 3.11	6.34 ± 3.80	0.860	
Number of spectators	0	1828.08 ± 1130.53	0	2180.15 ± 1236.37	< 0.001	G1 vs. G2, G1 vs. G4, G2 vs. G3, G3 vs. G4 (*p* < 0.001)

Values are presented as mean ± standard deviation.

Group definition: G1 = Home without spectators, G2 = Home with spectators, G3 = Away without spectators, G4 = Away with spectators.

### Data collection

To evaluate overall team performance, match data were retrospectively obtained from official records produced and disseminated by the Korea Professional Football Championship League. The collected variables included average points, goals scored, goals conceded, goal difference, ball possession, total shots, shots on target, fouls committed, fouls drawn, yellow and red cards. These variables were categorized into three domains based on their conceptual relevance. Outcome factors, including points, goals scored, goals conceded, win percentage, and goal difference, represent the final results of each match and serve as direct indicators of competitive success. Tactical factors, including ball possession, total shots, and shots on target, reflect the tactical execution and offensive engagement exhibited by teams during gameplay, irrespective of the final result. Violation factors, including fouls committed, fouls drawn, yellow cards, and red cards, capture the level of physical contact and disciplinary actions, thereby offering insights into match intensity and referee behavior. This classification enabled a structured analysis of both the results and the underlying performance characteristics.

### Statistical analysis

Data averages were compared and analyzed using the independent samples t-test and chi-square test. Group comparisons were conducted using one-way analysis of variance, followed by *post hoc* analysis with Tukey’s honestly significant difference test or Dunnett’s T3 test, depending on the homogeneity of variances. For each variable, the magnitude of pairwise differences was further assessed using Cohen’s *d*-value, which was interpreted as follows: < 0.20, negligible; 0.20–0.49, small; 0.50–0.79, medium; and ≥ 0.80, large (Rosenthal, 1996). Statistical significance was defined as a *p*-value < 0.05. All statistical analyses were performed using IBM SPSS Statistics version 20 (IBM Corp., Armonk, NY, USA).

### Ethics

The study protocol was approved by the Institutional Review Board of the Korea National Institute for Bioethics Policy (approval number: P01-202111-21-021), which waived the need for informed consent was waived during the deliberation process. This study complied with the principles of the Declaration of Helsinki.

## Results

**Figure 2:**
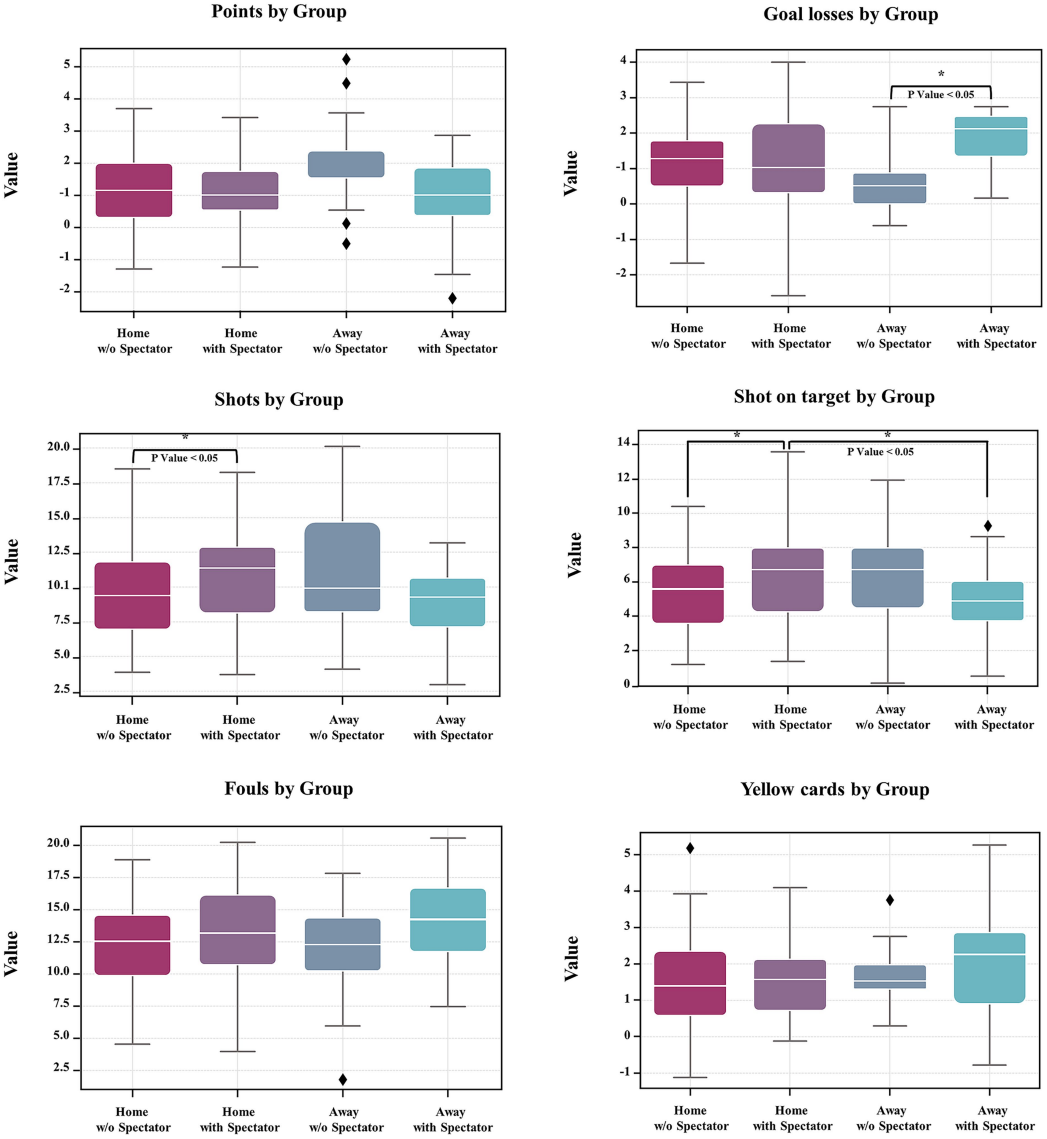
Boxplots comparing match performance metrics across four match types categorized by spectator presence and venue.

The presence of spectators was associated with improved home team performance, particularly in home matches. Specifically, tactical performance indicators, such as shooting activity, were significantly improved when home crowds were present. Home matches with spectators demonstrated significantly higher numbers of shots on target (6.02 ± 3.08) compared to both spectator-free home matches (4.40 ± 2.34; *p* = 0.041, *d* = 0.58) and away matches with spectators (4.48 ± 2.61; *p* = 0.017, *d* = 0.55). Likewise, the total number of shots was significantly greater in home matches with spectators (11.46 ± 4.13) than in home matches without spectators (9.46 ± 3.48; *d* = 0.52) and in away matches with spectators (8.93 ± 3.52; *p* = 0.005, *d* = 0.67). Conversely, the presence of spectators in away matches was associated with deteriorated outcome and violation performance indicators. Teams conceded significantly more goals in away matches with spectators (1.58 ± 1.16) than those without spectators (0.67 ± 0.89; *p* = 0.032, *d* = 0.82). Although not statistically significant, the highest number of yellow cards was seen in away matches with spectators (2.13 ± 1.36), with a small effect (*d* = 0.49) (Table 2; Figure 2).

**Table 2:** Comparison of match performance across home and away games with and without spectators.

Variables	Home matches		Away matches		*p* value	*Post-hoc* significance
	Without spectators (*n* = 35)	With spectators (*n* = 48)	Without spectators (*n* = 12)	With spectators (*n* = 67)		
Outcome factors						
Points	1.23 ± 1.29	1.31 ± 1.32	2.00 ± 1.28	0.96 ± 1.19	0.054	
Goals	0.94 ± 0.94	1.25 ± 1.00	1.08 ± 0.79	1.09 ± 1.18	0.625	
Goal conceded	1.09 ± 1.07	1.38 ± 1.21	**0.67 ± 0.89**	**1.58 ± 1.16**	**0.032**	**G3 vs G4 (*p* = 0.032)**
Win percentage	0.31 ± 1.24	0.35 ± 0.48	0.58 ± 0.52	0.22 ± 0.42	0.073	
Goal difference	−0.14 ± 1.24	−0.13 ± 1.50	0.42 ± 1.08	−0.49 ± 1.59	0.185	
Tactical factors						
Ball possession	49.49 ± 7.90	49.65 ± 9.03	48.67 ± 7.49	47.12 ± 9.36	0.417	
Shots	9.46 ± 3.48	**11.46 ± 4.13**	10.50 ± 4.23	**8.93 ± 3.52**	**0.005**	**G2 vs G4 (*p* = 0.005)**
Shots on target	**4.40 ± 2.34**	**6.02 ± 3.08**	6.50 ± 2.91	**4.48 ± 2.61**	**0.003**	**G1 vs G2** **(*p* = 0.046)** **G2 vs G4** **(*p* = 0.034)**
Violation factors						
Fouls	11.69 ± 3.45	13.33 ± 3.82	12.42 ± 3.68	13.16 ± 3.43	0.156	
Fouls drawn	12.34 ± 3.57	13.33 ± 3.81	10.92 ± 3.68	13.43 ± 4.15	0.138	
Yellow cards	**1.63 ± 1.37**	**1.52 ± 1.17**	**1.50 ± 0.91**	**2.13 ± 1.36**	**0.047**	
Red cards	0.11 ± 0.32	0.08 ± 0.28	0.00 ± 0.00	0.06 ± 0.30	0.636	

Values are presented as mean ± standard deviation.

Values in bold indicate statistically significant differences (*p* < 0.05).

Group definition: G1 = Home without spectators, G2 = Home with spectators, G3 = Away without spectators, G4 = Away with spectators.

**Figure 3:**
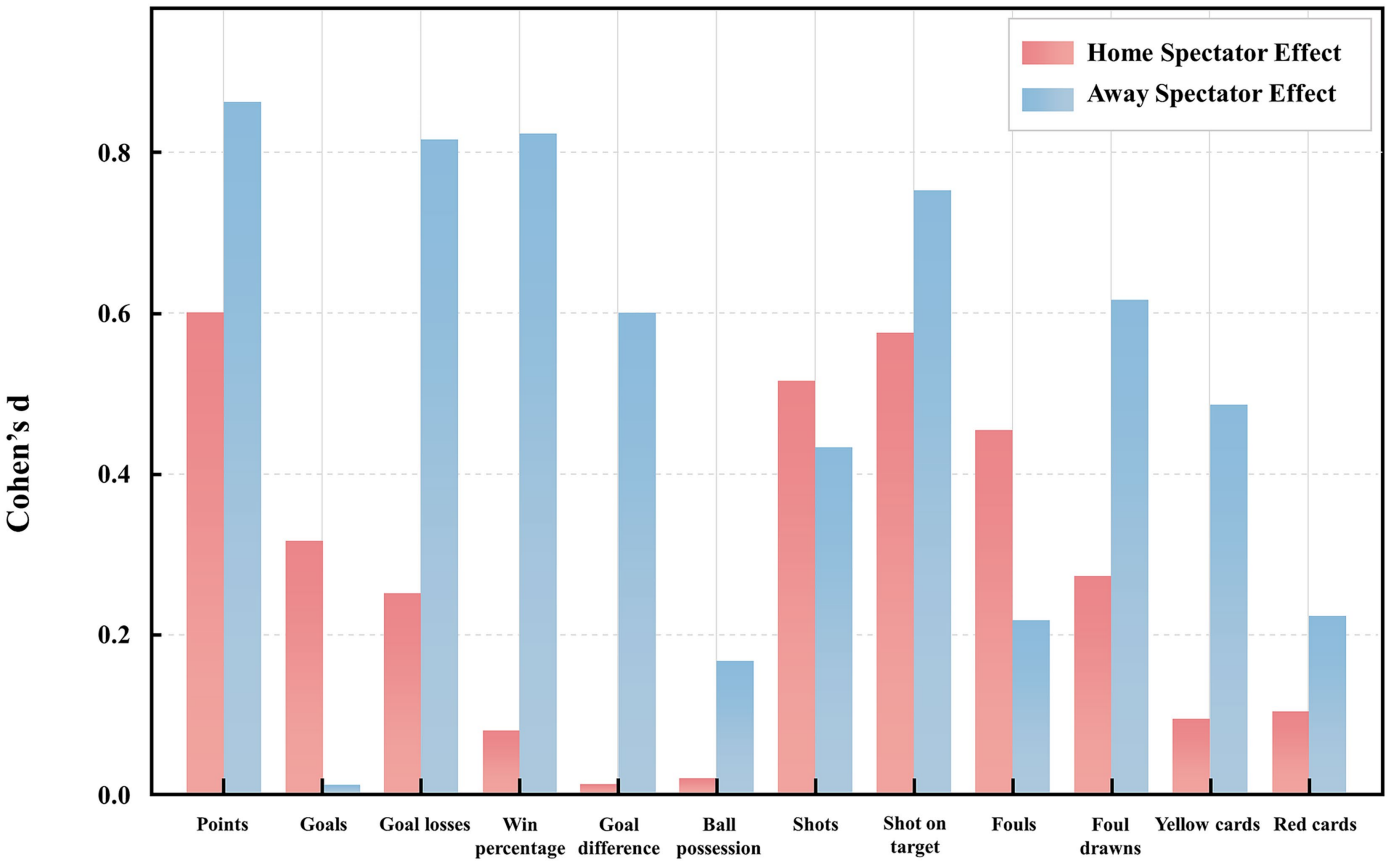
Comparison of Cohen’s *d* effect sizes for spectator presence during home and away matches.

Spectator-related effects were more pronounced in away matches than in home matches, particularly in outcome performance factors. Teams competing in away matches with spectators recorded a significantly lower win percentage (0.22 ± 0.42) than those without spectators (0.58 ± 0.52), with a large effect size (*d* = 0.83) (Table 3; Figure 3). Similarly, they conceded more goals (1.58 ± 1.16 vs. 0.67 ± 0.89, p = 0.032, *d* = 0.82), had a poorer goal difference (−0.49 ± 1.59 vs. +0.42 ± 1.08, *d* = 0.60), earned achieved fewer points (0.96 ± 1.19 vs. 2.00 ± 1.28, *d* = 0.87) and had fewer shots on target (4.48 ± 2.61 vs. 6.50 ± 2.91, *d* = 0.76). By comparison, in home matches, the presence of spectators was linked to increased shot attempts (9.46 ± 3.48 vs. 11.46 ± 4.13, *d* = 0.52) and shots on target (4.40 ± 2.34 vs. 6.02 ± 3.08, *p* = 0.046, *d* = 0.58). However, no significant differences were found in goals scored (0.94 ± 0.94 vs. 1.25 ± 1.00, *d* = 0.32), win percentage (0.31 ± 0.47 vs. 0.35 ± 0.48, *d* = 0.08), or goal difference (−1.40 ± 1.24 vs. -0.13 ± 1.50, *d* = 0.01) (Table 3; Figure 3).

**Table 3:** Pairwise Cohen’s *d* effect sizes between groups.

Variables	G1 vs. G2	G1 vs. G3	G1 vs. G4	G2 vs. G3	G2 vs. G4	G3 vs. G4
Outcome factors						
Points	**0.60**	0.22	**0.60**	0.29	**0.52**	**0.87**
Goals	0.32	0.17	0.16	0.15	0.17	0.01
Goal conceded	0.25	0.18	0.41	0.18	**0.61**	**0.82**
Win percentage	0.08	0.21	**0.56**	0.29	0.47	**0.83**
Goal difference	0.01	0.24	0.47	0.24	0.38	**0.60**
Tactical factors						
Ball possession	0.02	0.27	0.11	0.27	0.11	0.17
Shots	**0.52**	0.15	0.28	**0.67**	0.23	0.43
Shots on target	**0.58**	0.03	**0.84**	**0.55**	0.16	**0.76**
Violation factors						
Fouls	0.45	0.43	0.21	0.05	0.24	0.22
Fouls drawn	0.27	0.28	0.40	0.03	**0.64**	**0.62**
Yellow cards	0.09	0.37	0.10	0.48	0.02	0.49
Red cards	0.10	0.18	0.41	0.08	0.33	0.22

Cohen’s *d* interpretation: < 0.2 negligible, 0.2–0.49 small, 0.5–0.79 medium, ≥ 0.8 large.

Bold values indicate *d* ≥ 0.5 (medium or large effect size).

Group definition: G1 = Home without spectators, G2 = Home with spectators, G3 = Away without spectators, G4 = Away with spectators.

## Discussion

Although the concept of home advantage has been widely acknowledged in prior research, the specific contribution of spectator presence remains elusive, largely because of the inability to manipulate audience attendance under controlled conditions (Loughead et al., 2011; Goumas, 2014a, 2014b; Ponzo and Scoppa, 2016; Hill and Van Yperen, 2021; McCarrick et al., 2021; Scoppa, 2021; Sors et al., 2021; Wunderlich et al., 2021). By analyzing matches conducted during a relatively stable phase of the COVID-19 pandemic, our study minimized temporal and contextual variability, enabling a more precise assessment of spectator influence. Our study’s primary finding was that spectator presence was associated with improved tactical performance, particularly among home teams, as reflected by higher numbers of shots and shots on target. However, outcome-related indicators, such as points and win percentages, remained unchanged.

Our findings confirm our hypothesis that the presence of spectators in home matches is associated with a measurable enhancement in team performance. Crowd noise can intensify offensive efforts among home teams and influence referees to penalize away teams more frequently (McCarrick et al., 2021; Sors et al., 2021). Spectator presence has also been linked to increased shooting activity and greater assertiveness in gameplay by home teams (McCarrick et al., 2021; Scoppa, 2021; Wunderlich et al., 2021). Consistent with these reports, our data revealed improvements in tactical performance, particularly in total shots and shots on target in the presence of spectators. Previous research has attributed these effects to heightened adrenaline levels and increased focus resulting from crowd support, which collectively contribute to enhanced tactical performances (Villa and Lozano, 2016; Huang et al., 2020). However, outcome variables, such as points (*d* = 0.06) and win percentage (*d* = 0.08), demonstrated negligible effect sizes and did not reach statistical significance. This discrepancy suggests that while spectators may motivate more active play, tactically assertive play does not always lead to favorable outcomes. Rather than reflecting a superficial reaction to crowd presence, these behavioral shifts likely stem from heightened psychological arousal and motivational drive induced by the audience, prompting players to adopt more aggressive and purposeful playing styles (Huang et al., 2020). Several recent studies have examined these crowd effects during the COVID-19 period (Bryson et al., 2021; Benz and Lopez, 2023). Bryson et al. analyzed matches across 17 countries and 23 leagues, finding that the absence of spectators reduced home advantage and significantly diminished referee bias against away teams (Bryson et al., 2021). However, their sample excluded South Korea and other Asian leagues. Benz and Lopez et al. applied bivariate Poisson regression to similar research questions and found that while the presence of fans consistently affected officiating and game dynamics, these effects were heterogeneous across leagues and shaped by cultural context (Benz and Lopez, 2023). Our study complemented this body of work by providing region-specific evidence from the K League and empirically capturing culturally contingent crowd effects in an Asian professional football league. By focusing on a stable phase within a single season, our study further isolates spectator-related effects from broader pandemic-induced disruptions, thereby contributing to the refinement of this research field. Our findings align with recent studies that emphasize the importance of crowd composition, not just presence (Humphreys et al., 2024; Colella et al., 2024). One study reported that home and away fans can exert opposing effects on match outcomes (Humphreys et al., 2024; Colella et al., 2024). In contrast, another study demonstrated that banning only away supporters significantly deteriorates the team performance of visiting teams (Humphreys et al., 2024; Colella et al., 2024). In our setting, only home spectators were allowed during the COVID-19 pandemic, enabling us to examine the consequences of such asymmetric crowd presence. The observed reduction in away team outcome variables in the presence of spectators suggested that the absence of moral support from away fans may disrupt tactical execution and exacerbate psychological pressure, thereby impairing competitive balance. However, our study diverges from earlier literature in one key area: the relation between spectator presence and fouls committed. Although previous studies have suggested that spectators either reduce overall fouls, increase yellow cards against away teams, or exert no significant effect, our findings demonstrate no significant difference in total fouls according to spectator presence (Goumas, 2014a; Hill and Van Yperen, 2021; Reade et al., 2021; Sors et al., 2021). However, yellow card issuance was highest in away matches with spectators, partially aligning with prior investigations that have shown home crowds may influence referees to penalize visiting teams more harshly (Goumas, 2014a; Hill and Van Yperen, 2021). This tendency for crowd-induced officiating bias has also been documented in non-pandemic eras, suggesting that such effects are not unique to the COVID-19 period (Pettersson-Lidbom and Priks, 2010; Singleton et al., 2023). This suggests that although the frequency of fouls remained stable, the disciplinary responses of referees may have been modulated by crowd dynamics. Therefore, our findings underscore the psychological and tactical impacts of spectators in home matches. Although the presence of spectators did not yield immediate improvements in match outcomes, it significantly increased offensive engagement, particularly in shooting behavior. These results offer empirical support for the spectators’ performance-enhancing roles and reinforce the multifaceted nature of home advantage in professional football.

Our findings also confirmed the second hypothesis that spectators’ influence was more pronounced in away than in home matches. Although both the home and away teams were affected by the presence of spectators, the magnitude of team performance change was more marked in distant contexts. This finding was consistent with the theoretical framework, suggesting that home spectators impose more significant psychological pressure on visiting teams, which can potentially disrupt their tactical execution and increase stress levels (Hill and Van Yperen, 2021; Sors et al., 2021). This asymmetric influence was supported by large effect sizes in away matches with spectators: the win percentage dropped significantly (*d* = 0.83), and goal difference (*d* = 0.60), points earned (*d* = 0.87), and shots on target (*d* = 0.76) all declined compared to away matches without spectators. By contrast, the corresponding effect sizes in home matches were smaller (win percentage: *d* = 0.08, goals: *d* = 0.32, goal difference: *d* = 0.04, points: *d* = 0.60, shots on target: *d* = 0.58). These findings suggested that spectator presence predominantly magnifies the disadvantages faced by away teams, rather than equally enhancing home team advantages. Unlike previous studies that predominantly relied on ratio-based assessments or aggregated season-long data to evaluate home advantage (Hill and Van Yperen, 2021; McCarrick et al., 2021; Scoppa, 2021; Sors et al., 2021), our study directly compared match-level team performance encompassing outcome, tactical, and violation factors within a uniform COVID-era dataset. This methodological design minimizes confounding from inter-seasonal variability, rule modifications, or exogenous disruptions. Furthermore, by focusing exclusively on matches conducted under consistent public health protocols, we could more accurately isolate the impact of spectator presence, independent of broader pandemic-related factors. Therefore, although spectators enhanced active tactical execution in home teams, their presence had a more pronounced and detrimental impact on away teams, particularly in outcome-related metrics. This asymmetry suggests that while spectatorship contributes to enhancing home team efforts, its greater influence may lie in disrupting away team performance and stability, offering a more nuanced understanding of the home advantage.

Our study has several limitations. First, although the presence of spectators was a key variable, the actual number of attendees was significantly restricted due to ongoing public health regulations during the 2021 season. Stadium capacity was limited to 10–30%, potentially attenuating the full psychological and behavioral impact of crowd support (Jung, 2021) (see Supplementary material). This constraint may have led to an underestimation of the true magnitude of spectator effects under normal full-capacity conditions. Second, our dataset was limited to 162 matches within a single season. This relatively small sample size—compared to multi-season or multi-league studies—was necessary to maintain consistency in epidemiological and competitive conditions (Hill and Van Yperen, 2021; McCarrick et al., 2021; Scoppa, 2021; Sors et al., 2021; Wunderlich et al., 2021). However, this restriction may have reduced statistical power and limited the scope for subgroup analyses. Third, an imbalance existed in spectator access across regions. Non-capital region teams were permitted to host spectators for longer periods than teams in the capital region, resulting in a higher number of away matches with crowd presence. This structural asymmetry may have introduced bias in estimating the effects of spectatorship, particularly in outcome variables. Fourth, the nature of football itself imposes analytic limitations. The low-scoring structure and discrete point system (0, 1, or 3 points per match) inherently limit the variability of outcome measures, making it more difficult to detect statistically significant differences. Fifth, this study did not attempt to assess whether the home advantage had returned to pre-pandemic levels following the return of spectators. Although some prior research has reported a resurgence of home advantage post-pandemic, our analysis was not designed to evaluate longitudinal recovery (van Ours, 2024). Instead, we focused on immediate behavioral and performance effects within a single season under relatively stable crowd policies. Sixth, although prior studies have also examined spectator effects in other sports, such as basketball, baseball, handball, volleyball, swimming, and darts, their applicability to our research context remains limited (Losak and Sabel, 2021; Szabó, 2022; Chowdhury et al., 2024; Mcfall and Whitehead, 2024; Spilker and Ötting, 2024). Notably, Colella et al. reported that the impact of moral support from spectators becomes more pronounced when the levels of the competing teams are closely matched (Colella et al., 2024). Given that football is characterized by low-scoring matches where narrow margins frequently determine outcomes, it represents an ideal setting for evaluating the influence of spectator presence under conditions of delicate performance balance. Furthermore, unlike other sports that experienced prolonged or uniform spectator bans during the pandemic, football—especially in the context of the 2021 K League—offered a unique opportunity for within-season comparisons under regionally variable crowd policies. For these reasons, football was selected as the focus of our investigation. Nonetheless, our findings may not be readily generalizable to other sports due to structural and contextual differences in gameplay and audience dynamics. Finally, to the best of our knowledge, no prior peer-reviewed studies have examined the effects of spectator presence or absence using K League data. Previous large-scale analyses explicitly excluded South Korea and other Asian leagues (Bryson et al., 2021). Our study therefore fills a critical geographic and cultural gap, offering the first empirical evidence from Korean professional football. This contribution is significant, given recent findings that highlight the heterogeneity of spectator effects across leagues and cultural contexts (Benz and Lopez, 2023). From a strategic management perspective, our findings underscore the significant impact of crowd dynamics on shaping team behavior and competitive outcomes in professional football. To mitigate the adverse psychological effects of hostile spectator environments, particularly during away matches, teams should implement targeted psychological resilience programs. Additionally, incorporating crowd simulation drills into pre-match preparations may enhance players’ adaptability under pressure. These interventions could collectively reduce performance deterioration in away settings. At the policy level, league authorities should reconsider the current spectator allocation framework to promote a more equitable and competitively balanced environment.

## Conclusion

Our study demonstrates that the presence of spectators has a significant influence on team performance during professional football matches. Among home teams, spectator attendance was associated with a modest enhancement in tactical performance, as reflected by increased total shots and shots on target, suggesting a meaningful elevation in strategic intent and psychological arousal. In contrast, the presence of spectators in away matches had more pronounced effects, leading to a decrease in win percentage and an increase in goals conceded. This asymmetry suggests that although spectators may enhance home team performances, its most pronounced effect lies in undermining visiting teams by intensifying psychological pressure and hindering effective execution. These findings support the hypothesis that spectators contribute asymmetrically to home advantage, offering moderate benefits to home teams but more substantial adverse effects on away teams. Therefore, when evaluating offensive strategy, competitive fairness, and contextual match conditions in professional football, the distribution and composition of spectators should be explicitly considered.

## Data Availability

Publicly available datasets were analyzed in this study. This data can be found at: https://www.kleague.com/index.do.
