# Supplementary figures and images for: Asymmetric effects of spectator presence on home and away team performance: a natural experiment from the 2021 K League 1 season
Source: Front Psychol. 2025 Sep 1;16:1646264. doi: 10.3389/fpsyg.2025.1646264 (PMC12434956; doi:10.3389/fpsyg.2025.1646264)

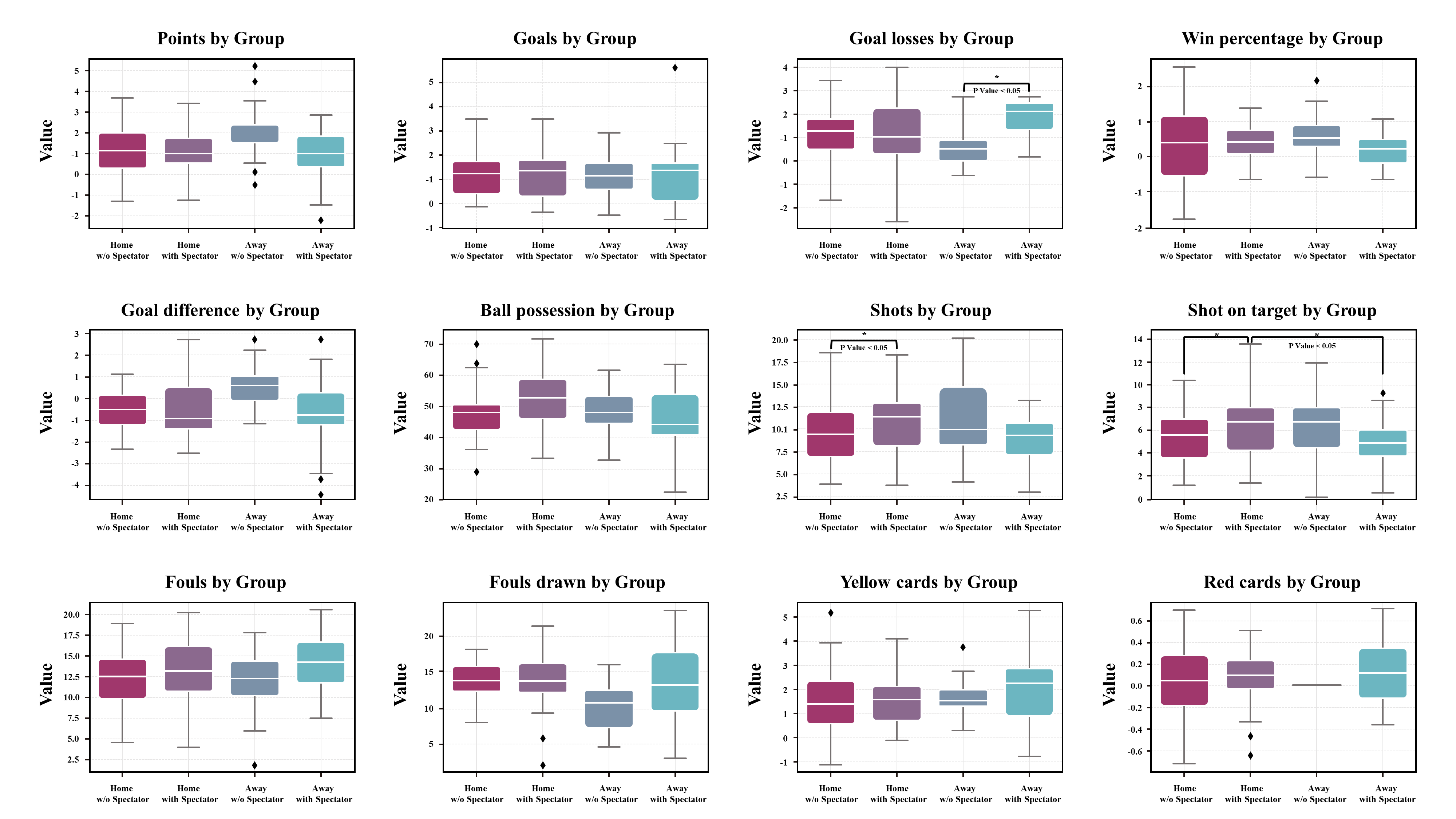

Supplement: Supplementary file 1 [file Image_1.tif]
